# Seroprevalence of Human Brucellosis among Syrian Refugees in Jordan, 2022

**DOI:** 10.1155/2023/5885316

**Published:** 2023-12-16

**Authors:** Tarek Al-Sanouri, Yousef Khader, Ekhlas Hailat, Sereen Iweir, Mohammad Abu Khudair, Mohannad Al Nsour

**Affiliations:** ^1^The Eastern Mediterranean Public Health Network, Amman, Jordan; ^2^Department of Public Health, Jordan University of Science and Technology, Irbid, Jordan

## Abstract

**Introduction:**

Brucellosis is prevalent in Mediterranean countries. The aim of this study was to determine the seroprevalence of brucellosis and associated factors among Syrian refugees in Jordan.

**Methods:**

A cross-sectional study was conducted among adult Syrian refugees who attended the Public Health Lab (PHL) in Al Mafraq governorate, during the period of May-June 2022 to obtain a health certificate, which is legally required to receive governmental authorization for employment in Jordan. Blood samples were obtained from participants and a serum specimen was tested for the presence of IgG antibodies against *Brucella* using enzyme-linked immunosorbent assay (ELISA) IgG kits (Vircell Microbiologists, Granada, Spain).

**Results:**

A total of 1562 Syrian refugees were enrolled in the study. Their ages ranged between 18 and 74 years, with a median age of 30 years at presentation. The majority were males (75.9%, *n* = 1186) and 24.1% (*n* = 376) were females. The *Brucella* ELISA IgG results were positive for 149 persons, with an overall seroprevalence rate of 9.5% (95% confidence interval: 8.0%–11.0%). Having animal-related occupations, residing outside refugee camps, consuming unpasteurized milk, handling animals or their tissues, and slaughtering animals within 6 months of study inclusion were significantly higher among the seropositive group. In the multivariate analysis, IgG-positive persons were 13 times more likely to report being diagnosed with brucellosis (OR = 13.1, 95% CI: 6.1–28.3; *p* ≤ 0.001). In addition, they were more likely to reside in the city of Al Mafraq, as opposed to a refugee camp (OR = 1.9, 95% CI: 1.1–3.2; *p* = 0.025) and to have handled animals within 6 months of study inclusion (OR = 3.1, 95% CI: 1.1–8.9; *p* = 0.035).

**Conclusions:**

In conclusion, one-tenth of adult Syrian refugees were tested positive for *Brucella* ELISA IgG. Being diagnosed with brucellosis, residing in the city of Al Mafraq, as opposed to a refugee camp, and handling animals within 6 months of study inclusion were significantly associated with being positive for *Brucella* ELISA IgG. This study illustrates the need for improved brucellosis control measures via comprehensive vaccination of animals and enhanced laboratory detection and surveillance capacities, in addition to emphasizing the need for increased awareness sessions among Syrian refugees on the safe use and preparation of dairy products and safety practices of handling animals and their tissues.

## 1. Introduction

Brucellosis is a bacterial endemic zoonotic disease of global significance with detrimental impacts on public health and food animal production. It is mainly transmitted to humans either through contact with infected animals (typically small ruminants and bovines) or due to consumption of unpasteurized dairy products as bacteria sheds in milk and other discharges [1]. In humans, brucellosis as an illness is debilitating, and its symptoms, including fever, sweating, fatigue, weight loss, headache, and joint pain may persist for weeks to months [2, 3]. In domestic animals, the disease causes significant livestock production losses resulting in major consequences on the economies of affected countries [4]. Furthermore, *Brucella* is being increasingly recognized for its potential use as a biological weapon [5].

The burden of the disease is more pronounced in developing countries due to inadequate surveillance and control measures, reduced domestic and imported animal-based control programs, and a lack of suitable diagnostic services. The highest incidence rates of brucellosis have been reported in Mediterranean countries [6], including Jordan. However, there is little information on the risk factors and geographical patterns of human brucellosis in Jordan and its characterization among refugees.

In spite of the considerable progress of knowledge gained and success achieved in brucellosis control in the developed world, this disease continues to be an important burden in the Middle East [1]. Over the last decade, an average of about 145 brucellosis cases was reported by the Ministry of Health (MOH) annually in Jordan, with the incidence rate of brucellosis almost doubling from the ten-year period of 2010 to 2020 (2.1 to 4.5 per 100,000 population) [1, 7]. However, the disease tends to be universally underreported [1]. Interestingly, in all published reports, Karak and Al Mafraq governorates accounted for over 50% of reported cases, despite representing less than 9% of the total population of Jordan [1, 7].

Data on seroprevalence in Jordan and Syria are limited and old. The only study in Jordan was conducted in 1992 among high-risk individuals in the north of Jordan. The study revealed a significantly higher seroprevalence of brucellosis among high-risk people (8.2%) compared to the control sample (0.5%). Seroprevalence among sheep farmers (12.5%) and meat handlers (4.9%) was significantly higher than that with other occupations [8]. On the other hand, studies conducted in Syria revealed that the seroprevalence of brucellosis in cattle ranged from 17.48% in 1992 to 2.59% in 1996, due to vaccination campaigns [9]. A nationwide cross-sectional study in Jordan showed that the estimated seroprevalence values were 18.1% (95% CI: 11, 25.3) in cattle herds, 22.2% (95% CI: 16.5, 28.8) in sheep flocks, 38.5% (95% CI: 24.3–51.8) in mixed herds of cattle and small ruminants, 45.4% (95% CI: 30.3, 61.6) in goat herds, and 70.4% (95% CI: 55.5, 84.9) in mixed sheep-goat flocks. The true prevalence across all small ruminant flocks was estimated as 34.3% (95% CI: 28.4, 40.4) [10]. Alarmingly, the World Organization for Animal Health's report revealed Syria to have the highest annual incidence rate of human brucellosis worldwide, reaching an annual rate of 1603 cases per million [11].

The high incidence of brucellosis in Jordan is noteworthy because Jordan has undergone significant socioeconomic shifts due to the recent conflict in the neighboring country and the consequent influx of around 1.3 million Syrian refugees since 2013 [12]. The conflict has been associated with an increased incidence of infectious diseases [13], including brucellosis in neighboring or host countries. In fact, cases of brucellosis among Syrian refugees are being increasingly detected in European countries [14, 15]. No studies have yet been conducted to estimate brucellosis incidence among Syrian refugees of Jordan, but previous reports have highlighted the increased smuggling of unvaccinated livestock along the Jordan-Syrian border in recent years [16]. In addition, the Al Mafraq Health Directorate's annual reports of human brucellosis cases within its governorate revealed that between the years of 2019 and 2021, an average of 132 cases were detected, of whom 38 patients were Syrian.

A cross-sectional study was conducted to estimate the seroprevalence of brucellosis and determine its associated factors among Syrian refugees in the Al Mafraq governorate in the north of Jordan, including residents of the governorate's refugee camp.

## 2. Methods

### 2.1. Study Setting

This study was conducted among Syrian refugees in the Al Mafraq governorate, including residents of the Zaatari refugee camp. The Zaatari refugee camp, located 10 km east of Al Mafraq city, is the world's largest camp for Syrian refugees and the second-largest refugee camp worldwide. As of 30 September 2023, Zaatari hosts 83,923 refugees in a 5.2 km^2^ area [17] and an additional 85,191 Syrian refugees reside in Al Mafraq governorate outside of Zaatari [17].

### 2.2. Study Design

This cross-sectional study included all Syrian refugees who attended the Public Health Lab (PHL) in Al Mafraq governorate during the period of May-June, 2022. They attended the Comprehensive Health Center to conduct routine blood examinations and to obtain a health certificate, which is legally required to receive governmental authorization for employment in Jordan. The PHL of Al Mafraq is the central public lab of the Al Mafraq Public Health directorate assigned to serve all residents of Al Mafraq. Eligible study subjects included any adults (≥18 years of age) arriving at the Public Health Lab (PHL) of Al Mafraq between May 31, 2022, and June 14, 2022, for any reason. Only those who had a verifiable identity card proving their Syrian nationality and refugee status were eligible for inclusion. The study team explained the purpose of the study to eligible subjects and a blood sample was withdrawn after they provided their written informed consent.

Assuming that the seroprevalence is 10%, the sample size needed to estimate the seroprevalence within a margin of error of 2% was calculated at 865 at a power of 80% and level of significance of 0.05. The sample size was calculated using Statulator [18].

### 2.3. Investigation Form

The study participants were interviewed by using a pretested structured questionnaire that was adapted from the previously validated investigative forms published by the United States Centers for Disease Control and Prevention (CDC) [19] and the official case report form of the Ministry of Health of Jordan. The questionnaire included questions regarding the age, sex, education, employment, exposure to animals, history of symptoms suggestive of brucellosis, history of established brucellosis diagnosis, and description of treatment received, if any. The investigation form included questions regarding certain characteristics or activities that have been reported as risk factors for brucellosis. These known risk factors included eating raw meat, consuming unpasteurized milk (or fresh nonboiled milk), consuming dairy products prepared from nonboiled milk, preparing dairy products at home, handling animals, animals' vaccination, handling animal tissues (placenta, aborted fetus, and birth fluids), and slaughtering/skinning animals within 6 months from the date of filling the investigation form.

The participants were asked if they experienced any of the following symptoms within 6 months of the date of filling the investigation form: undulant fever, night sweats, headache, joint pain, loss of appetite, malaise, myalgia, back pain, and depression.

The KoboToolbox application (Kobo Inc., Ontario, Canada) was used to create a data entry screen with necessary validation rules and skips. Android-operated tablets loaded with Kobo software were used for data collection. Data were synchronized daily with a secure FTP server at the Eastern Mediterranean Public Health Network (EMPHNET) offices to allow for repairing any data entry errors while data collectors were still in the field.

### 2.4. Lab Testing

At the PHL, sera samples were separated daily upon arrival and kept at 2–8°C until processed (within a maximum of three days of specimen collection), and each serum specimen was tested for the presence of IgG antibodies against *Brucella* using enzyme-linked immunosorbent assay (ELISA) IgG kits (Vircell Microbiologists, Granada, Spain). Universal biosafety and preventive precautions were considered during the withdrawal, transportation, and testing of blood specimens.

### 2.5. Data Analysis

Data analysis was conducted using SPSS version 24 (IBM, Armonk, New York, United States). Data were described using percentages. The chi-square test was used to compare background and *Brucella*-related variables between the ELISA IgG-positive and the ELISA IgG-negative subjects. Binary logistic regression analysis was conducted to assess the associations between studied variables and the ELISA IgG results. A *p* value of less than 0.05 was considered statistically significant.

## 3. Results

### 3.1. Participants' Characteristics

A total of 1562 Syrian refugees were enrolled in the study. Their ages ranged between 18 and 74 years, with a median age of 30 years at presentation. The majority were males (75.9%, *n* = 1186) and 24.1% (*n* = 376) were females. Of those, 20 (5.3%) were pregnant at the time of the study. The median family size was 5 members. Of all participants, 410 (26.3%) reported that they are currently employed. Of the employed participants, 78 (19.0%) reported having animal- or animal-tissue-related occupations such as shepherd, butcher, animal farm guardian, producing dairy products, working on poultry farms, and handling or packing meat products. Only two of the participants in this category were females (both working as farmhands on farms containing animals). When asked about their address, 303 (19.4%) of the participants revealed that they are residents of the Zaatari camp (Table 1).

### 3.2. Brucella-IgG Prevalence and Associated Characteristics

The *Brucella* ELISA IgG results were positive for 149 persons, resulting in an overall seroprevalence rate of 9.5% (95% confidence interval: 8.0%–11.0%). Of those, 21 (14.1%) reported being previously diagnosed with brucellosis, and 20 received treatment (rifampicin and doxycycline) after their diagnosis. The reported length of treatment ranged between 20 days and 2 years (42 days was the most frequent response (*n* = 7)). The ages of the seropositive participants also ranged from 18 to 74, with a median age of 33 years.

Overall, 38 (24.5%) of the seropositive participants were employed; 15 persons had animal-related occupations. The proportion of persons who reported having animal-related occupations was significantly higher among seropositive subjects than in seronegative subjects (*p* = 0.003). Only 13.4% of the IgG-positive cohort resided in a refugee camp compared to 21.7% of the IgG-negative participants (*p* = 0.009).

When asked if they recalled being previously diagnosed with the disease, 14.1% of the IgG-positive cohort reported a history of brucellosis diagnosis compared to only 1.0% of the IgG-negative participants (*p* ≤ 0.001). In addition, 5.8% of the IgG-positive and 0.7% of the IgG-negative participants had family members who were diagnosed with the disease (*p* ≤ 0.001) (Table 1).

Overall, 26.8% (*n* = 40) of the *Brucella* IgG-positive participants reported at least one of these characteristics: eating raw meat, consuming unpasteurized milk or fresh nonboiled milk, consuming dairy products prepared from nonboiled milk, preparing dairy products at home, handling animals, handling animal tissues (placenta, aborted fetus, and birth fluids), and slaughtering/skinning animals within 6 months from the date of filling the investigation form, compared to 14.4% (*n* = 203) of the IgG-negative participants (*p* ≤ 0.001). Consumption of unpasteurized milk, slaughtering animals, and handling their tissues were significantly more prevalent among the IgG-positive participants (Table 1).

### 3.3. Multivariate Analysis

In the multivariate analysis, IgG-positive persons were 13 times more likely to report being diagnosed with brucellosis (OR = 13.1, 95% CI: 6.1–28.3; *p* ≤ 0.001). In addition, they were more likely to reside in the city of Al Mafraq, as opposed to a refugee camp (OR = 1.9, 95% CI: 1.1–3.2; *p* = 0.025), and to have handled animals within 6 months of study inclusion (OR = 3.1, 95% CI: 1.1–8.9; *p* = 0.035) (Table 2).

### 3.4. Symptoms of Brucellosis

Table 3 shows the differences in participants' responses to symptoms questions according to the IgG-status. Among the symptomatic participants, the average number of symptoms displayed within 6 months of data collection was 6 symptoms for the IgG-positive cohort and 4.4 for the IgG-negative cohort. Undulant fever, headache, joint pain, and loss of appetite were significantly more prevalent among the seropositive group. Overall, the portion of those who experienced any of the abovementioned symptoms was significantly larger among the seropositive participants (6.0% vs 2.8%, *p* = 0.045) (Table 3). However, multivariate logistic regression analyses of the reported symptoms revealed that none of the symptoms were significantly associated with a positive *Brucella* IgG result (Table 4).

## 4. Discussion

This is the first study of brucellosis seroprevalence among a relatively large number of refugees in Jordan. The findings of this study portray the overall status of the seroprevalence of *Brucella* IgG among Syrian refugees in Al Mafraq and the governorate that hosts the largest population of Syrian refugees in Jordan, as well as containing the Zaatari camp.

The results revealed that close to 10% of the population might have a past brucellosis infection as evidenced by the detection of *Brucella* antibodies in their sera. This rate is higher than the rates reported in other areas such as Jammu in India (4.96%) [20], Laconia in Greece (8.0%) [21], and the Wadi Al Dawasir region of Saudi Arabia (8.6%) [22]. The findings of this study have significant implications for shaping government health policies on brucellosis. The findings highlight the need for enhanced surveillance and screening programs, not only for the refugee population but also for the host population. The government may consider expanding its testing efforts to identify and treat individuals with brucellosis more effectively. The government should evaluate and possibly expand its healthcare infrastructure to accommodate the increased demand for healthcare services resulting from a higher prevalence of brucellosis among the refugee population.

The analysis revealed that some variables were significantly associated with ELISA IgG results, particularly concerning the nature of their occupations, their locations, habitual exposure to, and slaughtering of livestock, as well as drinking unpasteurized milk.

Unpasteurized milk has long been considered the primary factor for brucellosis in humans [1, 2]. While only 18 participants in our cohort reported recent consumption of unpasteurized milk, six participants (33.3%) had positive *Brucella* IgG results (*p* = 0.005). Consumption of raw or unpasteurized dairy products and consumption of raw or undercooked meat was not significantly associated with seropositivity in this study. On the other hand, the seropositive cohort of the study population was significantly likely to have handled animals, primarily small ruminants (sheep in 82% of the cases) (OR = 3.1, 95% CI: 1.1–8.9; *p* = 0.035). These observations corroborate the trend observed in previous reports; whereby for lower to mid-income countries, brucellosis is more of a public health concern in terms of direct contact with livestock, rather than being a food-borne concern, as is the case of industrialized nations [23, 24]. This implies that any preventative actions or awareness activities to be conducted in Jordan may benefit from targeting animal-handling and occupational safety protocols and not solely focusing on food safety recommendations.

In this regard, a review of *Brucella* seroprevalence data in Bangladesh demonstrated that it was highest among those who had direct contact with animals, its products, and those who consumed raw milk. The seroprevalence was highest among livestock farmers (2.6%–21.6%), followed by milkers (18.6%), veterinarians (5.3%–11.1%), and butchers (2.5%) [25]. Similarly, many of the participants in our study revealed that they had occupations that involved direct contact with animals and their fluids/tissues (*n* = 78, 19.0%), with the higher portion observed among the seropositive group (10.5% vs. 4.5%, *p* = 0.003). Most frequently, those with animal-related occupations had agricultural professions, such as being a farmhand, or shepherd/herder (23 and 9 participants overall, respectively). This is in agreement with previous reports on agricultural occupations being the major sector of employment among Syrian refugees in Jordan [26]. The fact that 19.2% of those with animal-related occupations were IgG-positive, in addition to the seropositivity of 41.1% of the participants who handled animals highlights a need for increased awareness of the population working in the agricultural sector in the country, as it is possible that they are exposed to the disease in their jobs, which was observed in previous studies [27]. Interestingly, refugees living in refugee camps were less likely to be IgG-positive than the refugees living in the city of Al Mafraq and the governorate's neighboring towns, raising attention to the need for ensuring their inclusion in any brucellosis prevention, control, or awareness activities that may be conducted for refugees in the governorate.

Regarding the clinical presentation of the study participants, only 6% reported having any brucellosis symptoms within the last six months, with the rate being significantly higher among the seropositive group than that observed among the seronegative group. As *Brucella* IgG positivity is an indicator of a possible past infection or chronic case of brucellosis [28], seropositive participants might have exhibited the symptoms at an even earlier stage. Moreover, only 14.1% of the seropositive group recalled ever being diagnosed with the disease. This is a significant observation as it can be an indication that many of the brucellosis cases might have been misdiagnosed or undiagnosed. An investigation of 141, 604 lab-confirmed brucellosis cases in China by Wang et al. revealed that 57.6% of the patients had been misdiagnosed or suspected of having another disease, which was attributed to the absence of characteristic brucellosis symptoms and reduced awareness among physicians [29].

It is worth noting that the study has several limitations. For instance, the participants consisted of those arriving at the PHL of Al Mafraq on their own accord, which may have contributed to the relatively high portion of male participants (75.9%), in comparison to actual estimates that 51.6% of the overall population of Al Mafraq and 51% of the refugee population of Zaatari are males [30]. The study's focal points at the PHL reported that many of the participants came to the PHL to obtain a health certificate, which is legally required to receive governmental authorization for employment in Jordan. This also applies to the 303 refugees arriving from the Zaatari camp, as the local regulations in Jordan require that all residents and refugees in Al Mafraq can only obtain their official health certificates (whether employment or travel) from the PHL, despite them having access to public health centers and lab facilities at their camp. Reports have revealed that a minority of female Syrian refugees in Jordan join the labor force [31]. In our study, of the 332 female participants in this study, only 26 (7.8%) were employed. Future studies may thus benefit from utilizing a longer study duration, including a wider age group, and using an active sampling strategy to gain more comprehensive cohorts.

Nonetheless, as the first report of brucellosis seroprevalence among a large group of Syrian refugees, this study may be considered a baseline for future investigation of human brucellosis regarding refugees in Jordan and other at-risk populations in the country.

In conclusion, one-tenth of adult Syrian refugees tested positive for *Brucella* ELISA IgG. Being diagnosed with brucellosis, residing in the city of Al Mafraq, as opposed to a refugee camp, and handling animals within 6 months of study inclusion were significantly associated with being positive for *Brucella* ELISA IgG. Our study implies a need for increasing awareness among both the general population and the healthcare providers of Al Mafraq to address the apparent inefficient diagnosis of brucellosis, especially considering the importance of timely diagnosis for effective treatment of the illness and prevention of chronic disease. Ultimately, our results illustrate the need for improved brucellosis control measures via comprehensive vaccination of animals and enhanced laboratory detection and surveillance capacities, in addition to emphasizing the need for increased awareness sessions among Syrian refugees on the safe use and preparation of dairy products and safety practices of handling animals and their tissues.

## Figures and Tables

**Table 1:** The demographic and relevant characteristics of participants according to *Brucella* IgG test result.

Characteristics	Total *N* (%) (*N* = 1562)	IgG-positive *n* (%) (*N* = 149)	IgG-negative *n* (%) (*N* = 1413)	P value
*Sex*				0.365
Male	1186 (75.9)	118 (79.2)	1068 (75.6)	
Female	376 (24.1)	31 (20.8)	345 (24.4)	
*Age (year)*				0.134
18–39	1101 (70.5)	95 (63.8)	1006 (71.2)	
40–60	421 (27.0)	49 (32.9)	372 (26.3)	
>60	37 (2.4)	2 (1.3)	35 (2.5)	
*Employment status*				0.922
Unemployed	1150 (73.7)	111 (74.5)	1039 (73.6)	
Employed	410 (26.3)	38 (24.5)	372 (26.4)	
*Family size*				0.464
Single-member household	242 (15.5)	22 (14.8)	220 (15.6)	
2–5 members	372 (23.8)	38 (25.5)	334 (23.7)	
5–10 members	912 (58.4)	83 (55.7)	829 (58.6)	
>10 members	36 (2.3)	6 (4.0)	30 (2.1)	
*Residential area*				0.009
Outside the refugee camp	1233 (78.9)	129 (86.6)	1103 (78.0)	
Inside the refugee camp	329 (21.1)	20 (13.4)	309 (21.9)	
*History of brucellosis*	35 (2.2)	21 (14.1)	14 (1.0)	≤0.001
Having a family member with a previous diagnosis of brucellosis	17 (1.1)	8 (5.4)	9 (0.6)	≤0.001
*Engaged in the following activities in the last 6 months*				
Prepared dairy products at home	175 (11.2)	23 (15.4)	152 (10.8)	0.086
Handled animals	56 (3.6)	23 (15.4)	33 (2.3)	≤0.001
Slaughtered animals	32 (2.0)	12 (8.1)	20 (1.4)	≤0.001
Handled animal tissues	31 (2.0)	14 (9.4)	17 (1.2)	≤0.001
Ate raw meat	11 (0.7)	2 (1.3)	9 (0.6)	0.283
Consumed unpasteurized milk	18 (1.2)	6 (4.0)	12 (0.8)	0.005
Consumed raw or unpasteurized dairy products	33 (2.1)	4 (2.7)	29 (2.1)	0.388
Engaged in a known risk factor for brucellosis	243 (15.6)	40 (26.8)	203 (14.4)	≤0.001

**Table 2:** Univariate and multivariate analysis of factors associated with *Brucella* seropositivity.

Variable	Univariate analysis			Multivariate analysis		
	OR	95% CI	P value	OR	95% CI	P value
History of brucellosis (yes vs. no)	16.4	8.1–33.0	≤0.001	13.1	6.1–28.3	≤0.001
Having a family member with a previous diagnosis of brucellosis (yes vs. no)	8.8	3.3–23.2	≤0.001	2.8	0.8–10.0	0.107
Animal-related occupation (yes vs. no)	2.4	1.3–4.3	0.004	1.2	0.5–2.7	0.704
Residency place (outside the camp vs. inside the camp)	1.8	1.1–2.9	0.018	1.9	1.1–3.3	0.025
Handled animals within the last 6 months (yes vs. no)	7.6	4.3–13.4	≤0.001	3.1	1.1–8.9	0.035
Slaughtered within the last 6 months (yes vs. no)	6.1	2.9–12.7	≤0.001	1.1	0.3–3.6	0.910
Handled animal tissues within the last 6 months (yes vs. no)	8.5	4.1–17.6	≤0.001	2.6	0.8–8.9	0.129
Consumed unpasteurized milk within the last 6 months (yes vs. no)	4.9	1.8–13.2	0.002	1.1	0.3–4.6	0.873

**Table 3:** The difference in reported symptoms (within 6 months of the date of data collection) between the *Brucella* IgG-positive and IgG-negative cohorts.

Symptoms	IgG-positive (*N* = 149)	IgG-negative (*N* = 1411)	*P* value
Experienced any brucellosis symptoms	9 (6.0%)	40 (2.8%)	0.045
Undulant fever	8 (5.4%)	22 (1.6%)	0.005
Chills	3 (2.0%)	18 (1.3%)	0.444
Headache	8 (5.4%)	21 (1.5%)	0.004
Joint pain	8 (5.4%)	29 (2.1%)	0.020
Loss of appetite	5 (3.4%)	15 (1.1%)	0.035
Malaise	2 (1.2%)	17 (1.3%)	0.702
Myalgia	3 (2.0%)	19 (1.3%)	0.511
Back pain	4 (2.7%)	20 (1.4%)	0.278
Depression	2 (1.3%)	10 (0.7%)	0.320

**Table 4:** Univariate and multivariate analysis of signs and symptoms associated with *Brucella* seropositivity.

Symptoms	Univariate analysis			Multivariate analysis		
	OR	95% CI	P value	OR	95% CI	P value
Undulant fever	3.6	1.6–8.2	0.002	1.8	0.2–19.1	0.614
Headache	3.8	1.6–8.6	0.002	2.8	0.4–19.0	0.304
Joint pain	2.7	1.2–6.0	0.015	1.1	0.2–5.7	0.955
Loss of appetite	3.3	1.1–9.0	0.025	0.7	0.1–3.7	0.662

## Data Availability

The data used to support the findings of the study are available from the corresponding author upon request.

## References

[1] Bagheri Nejad R., Krecek R. C., Khalaf O. H., Hailat N., Arenas-Gamboa A. M. (2020). Brucellosis in the Middle East: current situation and a pathway forward. *PLoS Neglected Tropical Diseases*.

[2] Corbel M. J. (2006). *Brucellosis in Humans and Animals*.

[3] Kim J. W., Lee Y. J., Han M. Y. (2007). Evaluation of immunochromatographic assay for serodiagnosis of Brucella canis. *Journal of Veterinary Medical Science*.

[4] Kortepeter M. G., Parker G. W. (1999). Potential biological weapons threats. *Emerging Infectious Diseases*.

[5] Akakpo A. J., Têko-Agbo A., Koné P. (2009). The impact of brucellosis on the economy and public health in Africa. Compendium of technical items presented to the OIE World Assembly of Delegates or to OIE Regional Commissions. https://www.woah.org/fileadmin/Home/eng/Publications_%26_Documentation/docs/pdf/TT/2009_085-098_Akakpo_A.pdf.

[6] Hull N. C., Schumaker B. A. (2018). Comparisons of brucellosis between human and veterinary medicine. *Infection Ecology & Epidemiology*.

[7] Jordan Ministry of Health (2021). Annual communicable diseases surveillance report. https://doh.wa.gov/data-and-statistical-reports/diseases-and-chronic-conditions/communicable-disease-surveillance-data/annual-cd-surveillance-reports.

[8] Abo-Shehada M. N., Odeh J. S., Abu-Essud M., Abuharfeil N. (1996). Seroprevalence of brucellosis among high risk people in northern Jordan. *International Journal of Epidemiology*.

[9] Darwish M., Benkirane A. (2001). Field investigations of brucellosis in cattle and small ruminants in Syria, 1990-1996. *Revue Scientifique et Technique de l’OIE*.

[10] Musallam I., Abo-Shehada M., Omar M., Guitian J. (2015). Cross-sectional study of brucellosis in Jordan: prevalence, risk factors and spatial distribution in small ruminants and cattle. *Preventive Veterinary Medicine*.

[11] Pappas G., Papadimitriou P., Akritidis N., Christou L., Tsianos E. V. (2006). The new global map of human brucellosis. *The Lancet Infectious Diseases*.

[12] Breulmann M., van Afferden M., Al-Subeh A., Al-Mahamid J. S., Dorgeloh E., Müller R. A. (2021). National framework: the certification of wastewater treatment systems with capacities up to 5.000 PE in Jordan. https://www.researchgate.net/publication/353947058_National_Framework_The_Certification_of_Wastewater_Treatment_Systems_with_Capacities_up_to_5000_PE_in_Jordan.

[13] Petersen E., Baekeland S., Memish Z. A., Leblebicioglu H. (2013). Infectious disease risk from the Syrian conflict. *International Journal of Infectious Diseases*.

[14] Grunow R., Jacob D., Klee S. (2016). Brucellosis in a refugee who migrated from Syria to Germany and lessons learnt. *Euro Surveillance*.

[15] Isenring E., Fehr J., Gültekin N., Schlagenhauf P. (2018). Infectious disease profiles of Syrian and Eritrean migrants presenting in Europe: a systematic review. *Travel Medicine and Infectious Disease*.

[16] Hashemite Kingdom of Jordan Ministry of Planning and International Cooperation United Nations (2013). Host community support platform: needs assessment review of the impact of the Syrian crisis on Jordan. https://www.undp.org/sites/g/files/zskgke326/files/migration/arabstates/Jordan-Needs-Assessment---November-2013.pdf.

[17] Operational Data Portal (2018). Syria regional refugee response. https://data.unhcr.org/en/situations/syria/location/51.

[18] Dhand N. K., Khatkar M. S., Statulator an online statistical calculator (2014). Sample size calculator for estimating a single proportion. http://statulator.com/SampleSize/ss1P.html.

[19] Cdc (2023). Brucellosis case report form. https://www.cdc.gov/brucellosis/pdf/case-report-form.pdf.

[20] Sharma H. K., Kotwal S. K., Singh D. K. (2016). Seroprevalence of human brucellosis in and around Jammu, India, using different serological tests. *Veterinary World*.

[21] Andriopoulos P., Floros D., Gioti N., Mariolis A., Rojas Gil A. P., Tsironi M. (2018). Brucella seroprevalence in a high-risk population in Greece: a cross-sectional study. *Interdisciplinary Perspectives on Infectious Diseases*.

[22] Rahamathulla M. P. (2019). Seroprevalence of human brucellosis in Wadi Al dawaser region of Saudi Arabia. *Pakistan Journal of Medical Sciences*.

[23] Hald T., Aspinall W., Devleesschauwer B. (2016). World Health Organization estimates of the relative contributions of food to the burden of disease due to selected foodborne hazards: a structured expert elicitation. *PLoS One*.

[24] Godfroid J. (2017). Brucellosis in livestock and wildlife: zoonotic diseases without pandemic potential in need of innovative one health approaches. *Archives of Public Health*.

[25] Islam M. A., Khatun M. M., Werre S. R., Sriranganathan N., Boyle S. M. (2013). A review of Brucella seroprevalence among humans and animals in Bangladesh with special emphasis on epidemiology, risk factors and control opportunities. *Veterinary Microbiology*.

[26] Sahin Mencutek Z., Nashwan A. J. (2021). Perceptions about the labor market integration of refugees: evidences from Syrian refugees in Jordan. *Journal of International Migration and Integration*.

[27] Mangalgi S. S., Sajjan A. G., Mohite S. T., Gajul S. (2016). Brucellosis in occupationally exposed groups. *Journal of Clinical and Diagnostic Research*.

[28] Yagupsky P., Morata P., Colmenero J. D. (2019). Laboratory diagnosis of human brucellosis. *Clinical Microbiology Reviews*.

[29] Wang Y., Zhang W., Ke Y. (2013). Human brucellosis, a heterogeneously distributed, delayed, and misdiagnosed disease in China. *Clinical Infectious Diseases*.

[30] Foreign and Commonwealth Office (2013). *Zaatari Refugee Camp*.

[31] Chan L., Kantner M. (2019). Employment trends, challenges and opportunities for refugees in Jordan. https://www.alghurairfoundation.org/employment-trends-challenges-and-opportunities-for-refugees-in-jordan/.

